# Perceived Barriers to Care for Urinary Tract Infections in Emerging Adulthood

**DOI:** 10.1007/s11606-025-09565-9

**Published:** 2025-05-08

**Authors:** Jennifer Yarger, Anne M. Suskind, Ina U. Park, Iris Wong, Hannah K. Hecht, Cynthia C. Harper

**Affiliations:** 1 Philip R. Lee Institute for Health Policy Studies, University of California San Francisco, San Francisco, CA USA; 2 Department of Epidemiology and Biostatistics, University of California San Francisco, San Francisco, CA USA; 3 Department of Urology, University of California San Francisco, San Francisco, CA USA; 4 Department of Obstetrics, Gynecology and Reproductive Sciences, University of California San Francisco, San Francisco, CA USA; 5 Department of Family and Community Medicine, University of California San Francisco, San Francisco, CA USA

**Keywords:** Health services accessibility, Social determinants of health, Urinary tract infections, Young adult

## Abstract

**Background:**

Urinary tract infections (UTIs) are common in emerging adulthood, yet barriers to care are not well understood.

**Objective:**

To examine perceived barriers to UTI care among emerging adults, analyzing differences by social determinants of health.

**Design:**

Supplementary study to a cluster randomized controlled trial in 29 community colleges in California and Texas. Online surveys were administered May to October 2024. Multivariable mixed-effects logistic and linear regression were used to predict the most common barriers to UTI care and number of barriers by participant characteristics.

**Participants:**

A total of 667 individuals aged 19–29, assigned female at birth, and sexually experienced with male partners.

**Main Measures:**

Outcomes included 14 items assessing perceived barriers to UTI care, developed using the Levesque model of healthcare access as a framework, and a composite score of the total number of barriers.

**Key Results:**

The average age of participants was 22.6 years; 56% identified as Hispanic and 30% reported a prior UTI. The most common perceived barriers to UTI care were concerns about hearing bad news (59%), appointment delays (46%), cost (45%), fear of parents learning about symptoms (42%), time constraints (40%), and concerns that they might have a sexually transmitted infection (37%). On average, participants reported 4.18 barriers; in multivariable regression, participants reported significantly more barriers if they were younger (*β* =  − 0.19, SE = 0.06), Asian/Pacific Islander (*β* = 0.71, SE = 0.35), non-English language speakers at home (*β* = 0.57, SE = 0.25), food insecure (*β* = 1.32, SE = 0.24), uninsured (*β* = 0.65, SE = 0.28), or without a usual source of care (*β* = 0.97, SE = 0.23).

**Conclusions:**

Findings showed substantial challenges to UTI care among emerging adults, especially among socially disadvantaged participants. Youth-focused interventions, including education and expanded telehealth services, are needed to promote health equity for UTIs.

## INTRODUCTION

Urinary tract infections (UTIs) are among the most commonly treated outpatient infections, with particularly high incidence among sexually active young women.^1,2^ Approximately one-third of women will be treated for a UTI by age 24, and UTI-related health care costs exceed $2 billion per year.^1^ Acute uncomplicated UTIs contribute to pain and discomfort,^3,4^ and negatively impact daily activities, work productivity, mental health, and other quality of life indicators.^3–6^ If left untreated, an uncomplicated UTI can progress to pyelonephritis, also called a kidney infection, and life-threatening septic shock.^7^ Evidence suggests that women who perceive barriers to healthcare may delay or forgo needed care.^8,9^ Therefore, understanding perceived barriers to UTI care is essential for ensuring timely and appropriate care.

The existing literature points to multiple potential barriers to UTI care. Many individuals lack knowledge about UTI symptoms and treatments, which may influence their perceived need and desire for care.^10–12^ Additionally, feelings of embarrassment and stigma associated with urinary symptoms can prevent care-seeking,^4,13,14^ especially for symptoms that may be mistaken for sexually transmitted infections (STIs), which carry their own stigma.^15,16^ For those who desire care for UTI symptoms, less is known about the challenges in reaching or engaging in care. One study found that cost and long wait times influence where patients seek UTI care.^17^ Most of these studies are conducted in clinical settings, potentially excluding individuals who face the most difficulty accessing care. Researchers have called for more evidence on women’s experiences seeking care for urinary symptoms, including those not using health services.^13,18^

Studies have also focused predominantly on adult populations, leaving a gap in understanding emerging adults’ access to UTI care. Women ages 18–24 have the highest UTI incidence,^19^ and frequent sexual activity is a risk factor for acute pyelonephritis in this age group.^20^ Emerging adulthood is a critical stage when individuals learn to navigate the healthcare system independently and face unique barriers, such as low levels of health insurance literacy and aging out of health insurance coverage under their parent’s plan.^21–23^ In addition, young people under age 25 years have the highest STI rates^24^ and may experience greater stigma and shame related to STIs and sexual activity, affecting their willingness to seek care. Several qualitative studies examining women’s experiences with UTI care have included young adults, but none were specifically focused on this age group and all were conducted in European countries.^4^ There is a significant gap in the evidence on barriers to UTI care among emerging adults in the USA.

Despite widespread recognition of the importance of social determinants of health,^25–27^ empirical research on social determinants of health and UTI care remains limited. Two studies surveyed women in the UK about their UTI symptoms and care-seeking experiences but did not examine how care-seeking varied by background characteristics.^28,29^ One exception is a small study of adults hospitalized for UTIs in East Los Angeles, which found that non-English-speaking patients received less education from providers about UTIs and had lower UTI knowledge than English speakers.^30^ Research is needed to identify the social determinants of barriers to UTI care in the USA, so they can begin to be addressed.

This study examines perceived barriers to UTI care in a diverse sample of emerging adults recruited from community colleges in California and Texas. We use the Levesque model of healthcare access as a framework, which defines access as “the opportunity to reach and obtain appropriate healthcare services in situations of perceived need for care”.^31^ This model outlines steps patients navigate, from recognizing the need for care to fully engaging in a visit. We assess perceived barriers to UTI care at each step. Guided by a social determinants of health framework, we analyze differences in perceived barriers to UTI care based on health insurance, usual source of care, and key sociodemographic and socioeconomic characteristics. The findings can inform ways to ensure that emerging adults have access to timely and appropriate UTI care, minimizing pain and suffering, preventing more serious infections, and reducing costs to individuals and the health system.

## METHODS

This study is supplementary to an ongoing randomized controlled trial focused on contraceptive access among young people in community college, which is registered at ClinicalTrials.gov (NCT03519685). Launched in 2018, participants were recruited at 29 community college sites in California and Texas. At baseline, eligible participants were aged 18–25, assigned female at birth (gender inclusive), English speaking, had had vaginal sex with a male partner in the last year, were neither pregnant nor wanting to become pregnant, and were enrolled as a student at a community college site. All participants were given a written consent form and provided electronic consent to participate. Participants completed online surveys at baseline, quarterly for 1 year, and semi-annually for up to 4 more years for reproductive health, educational, and economic outcomes. Survey reminders were sent by email, text, social media direct message, or phone call.

In May 2024, we added a survey module on UTIs to follow-up surveys. The module included questions about perceived access to UTI care, recent UTI symptoms, self-treatment and at-home UTI testing, STI symptoms and testing, UTI diagnosis history, and recent UTI care. The authors developed the survey module based on a literature review and the subject-matter expertise of the author team. We pilot tested the survey items with 25 participants aged 18–25 assigned female at birth. This included conducting cognitive interviews to understand how respondents interpreted each item and gather feedback. We used the insights from these interviews to improve the clarity, reliability, and validity of the questions. The current analyses included surveys administered May to October 2024.

The Institutional Review Boards (IRBs) at the University of California, San Francisco, and the University of Texas at Austin approved the study; participating college sites approved the study with their IRB or used the corresponding state university’s IRB approval. Participants received a $20–$30 gift card after each follow-up survey.

### Measures

#### Perceived Barriers to Care for UTI Symptoms

We measured perceived barriers to care for UTI symptoms with 14 items in the survey module. The items addressed barriers to UTI care identified in qualitative studies.^4^ We also developed items to align with dimensions of abilities to access health care in the Levesque model, as shown in Table 1: ability to perceive the need for care, ability to seek care, ability to reach care, ability to pay for care, and ability to engage in care. In the cognitive interviews, we asked open-ended questions to ensure that we captured all relevant barriers to UTI care.

**Table 1:** Dimensions of Ability to Access Healthcare from the Levesque Model and Survey Items Measuring Perceived Barriers to Care for UTI Symptoms

Dimension of ability to access care	Survey item
Ability to perceive	I would know if I needed to seek care for urinary symptoms
Ability to seek	I would worry about hearing bad news
	I would worry that I might have a sexually transmitted disease
	I would worry about my privacy
	I would not want my parents to know about my urinary symptoms
Ability to reach	I would know where to go for care
	It would be easy to get to a clinic
	I would have to wait a long time for an appointment
	I would be too busy for an appointment
Ability to pay	I would worry about the cost
Ability to engage	I would feel comfortable discussing urinary symptoms with a doctor or nurse
	I would worry about a doctor or nurse judging me
	I would worry about trusting a doctor or nurse
	I would find it hard to talk to a doctor or nurse

Items were answered on 4-point Likert scale: strongly agree, agree, disagree, strongly disagree

The items were introduced with the following prompt: “The next questions ask about your ability to get medical care for urinary symptoms, such as frequent or painful urination, even if you have never experienced them. Please indicate if you agree or disagree with the following statements about getting medical care for urinary symptoms.” Items were answered on a 4-point Likert scale: “strongly agree,” “agree,” “disagree,” “strongly disagree.” We created dichotomous variables equal to 1 if the participant answered “strongly agree” or “agree” with each statement, and 0 for “disagree” or “strongly disagree.” We reverse-coded responses to four questions (about knowing when to seek care, where to go for care, ease of getting to a clinic, and feeling comfortable discussing urinary symptoms) so that all measures represent perceived barriers to UTI care. Additionally, we calculated a composite score by summing the 14 dichotomized items, with higher scores representing more barriers to care (range of scores 0–14, Cronbach’s alpha 0.75).

#### Participant Characteristics

To identify factors associated with perceived barriers to care for UTI symptoms, we examined two variables previously shown to be associated with healthcare access: whether the participant was uninsured (yes, no) and whether they lacked a usual source of care (yes, no). Sociodemographic variables included age in years, self-reported race and ethnicity (Hispanic, White non-Hispanic, Asian/Pacific Islander non-Hispanic, Black non-Hispanic, American Indian or Alaska Native/Multi-racial/other non-Hispanic; for brevity, Hispanic, White, Asian/Pacific Islander, Black, American Indian/Multi-racial/other), language spoken at home (English, language other than English), and baseline state of residence (California, Texas). We measured food insecurity (yes, no), using an item adapted from the USDA household food security module.^32,33^ Participants were asked how often their household worried about running out of food before they could buy more in the past month. They were coded as experiencing food insecurity if this was “often true” or “sometimes true.” We also included whether they had a prior UTI diagnosis (yes, no).

### Statistical Analysis

From the total sample of 682, we excluded observations for those who were missing data on any of the perceived barriers to UTI care (*n* = 5), any of the participant characteristics (*n* = 5), or both (*n* = 5), for a final analytical sample of 667.

First, we calculated the percentage of participants reporting each perceived barrier to UTI care. Next, we estimated multivariable mixed-effects logistic regression models with random effects for site to compare each of the six most common perceived barriers by sociodemographic and socioeconomic characteristics, health insurance, and usual source of care. Finally, we estimated a multivariable mixed-effects linear regression model, also with random effects for site, to examine the composite score of perceived barriers based on the same participant characteristics. Additionally, we conducted sensitivity analyses using multivariable mixed-effects ordered logistic regression to predict the outcomes treated as ordinal variables on the 4-point Likert scale. We also estimated a model predicting an alternate composite score of perceived barriers, calculated by summing the 14 ordinal items. Analyses were conducted in Stata version 18 and significance levels reported at *p* < 0.05.

## RESULTS

The participants had an average age of 22.6 years and were a racially and ethnically diverse group, with the largest proportion (56%) identifying as Hispanic (Table 2), which reflects the community college student populations in California and Texas.^34,35^ Access to healthcare also varied, with 36% lacking a usual source of care and 23% being uninsured. Half (50%) spoke a language other than English at home and 30% reported food insecurity. More than a quarter (30%) had been diagnosed with a UTI at some point.

**Table 2:** Participant Characteristics (*N* = 667)

	*n*	%
Age (years) (mean, SD)	22.6	1.9
Race and ethnicity		
Hispanic	377	56.5
White	131	19.6
Black	39	5.8
Asian/Pacific Islander	81	12.1
American Indian/multi-racial/other	39	5.8
Language spoken at home		
English	332	49.8
Non-English	335	50.2
Food insecure	200	30.0
Uninsured	153	22.9
No usual source of care	237	35.5
State		
Texas	279	41.8
California	388	58.2
History of UTI	200	30.0

*SD* standard deviation

**Figure 1:**
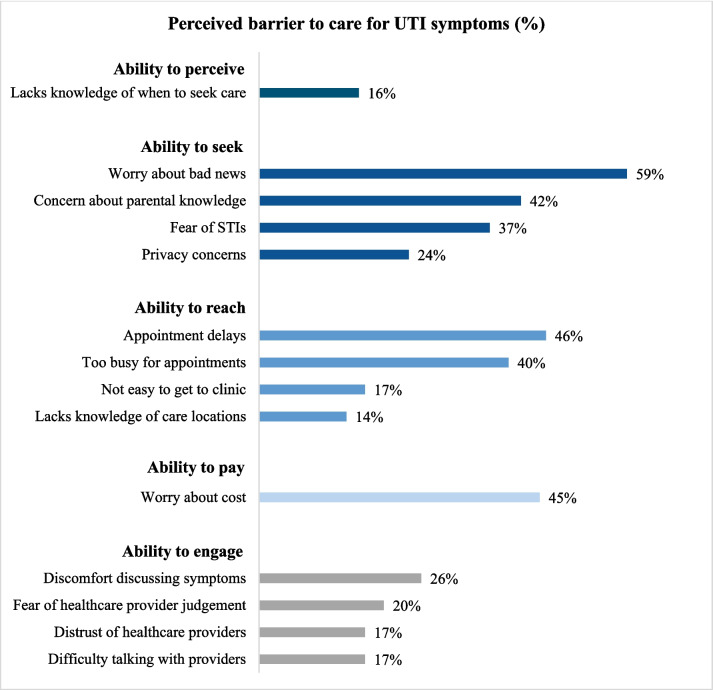
Perceived barriers to care for UTI symptoms, building on Levesque’s model of healthcare access (*N* = 667).

Participants reported barriers across multiple dimensions of access to UTI care, starting with recognizing a need for care (Fig. 1). Sixteen percent of participants reported that they would not know when to seek care for UTI symptoms. In terms of the ability to seek care, about one-quarter (24%) expressed concern about privacy and 42% would worry about their parents learning about their symptoms. Over half (59%) reported that they would worry about receiving bad news and 37% would worry that they might have an STI. Barriers to reaching care also were prevalent, with 46% anticipating appointment delays and 40% feeling too busy for appointments. Although less common, 14% did not know where to get care and 17% expressed concerns about getting to a clinic. Nearly half (45%) reported that they would worry about the cost of UTI care. Lastly, many participants perceived barriers to engaging in the visit, including discomfort discussing symptoms with providers (26%), fear of provider judgement (20%), distrust of healthcare providers (17%), and difficulty talking with providers (17%).

**Figure 2:**
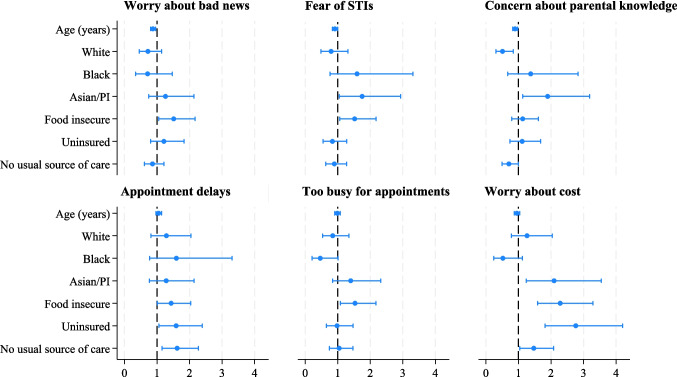
Adjusted odds ratios and 95% confidence intervals from multivariable mixed-effects logistic regression models predicting the most common perceived barriers to UTI care (*N* = 667). Note: PI, Pacific Islander. The reference category for race and ethnicity was Hispanic; results for American Indian/Multi-racial/other group not shown. All models controlled for state and history of UTI and included random effects for site.

Results of multivariable logistic regression models analyzing the most common perceived barriers to UTI care showed several factors to be associated with these barriers (Table 3, Fig. 2). In fully adjusted models, younger age was significantly associated with concerns about receiving bad news (adjusted odds ratio [aOR] = 0.88, 95% confidence interval [CI] 0.80–0.95), worries about having an STI (aOR = 0.91, 95% CI 0.84–1.00), and concerns about parents finding out about their symptoms (aOR = 0.90, 95% CI 0.82–0.98).

**Table 3:** Multivariable Mixed-Effects Logistic Regression Models Predicting the Most Common Perceived Barriers to UTI Care (*N* = 667)

	Worry about bad news		Fear of STIs		Concern about parental knowledge		Appointment delays		Too busy for appointments		Worry about cost	
	aOR	95% CI	aOR	95% CI	aOR	95% CI	aOR	95% CI	aOR	95% CI	aOR	95% CI
Age (years)	0.88**	0.80–0.95	0.91*	0.84–1.00	0.90*	0.82–0.98	1.05	0.96–1.14	0.99	0.91–1.08	0.95	0.87–1.04
Race and ethnicity												
Hispanic (Ref.)	1.00		1.00		1.00		1.00		1.00		1.00	
White	0.72	0.46–1.14	0.80	0.49–1.31	0.51**	0.31–0.84	1.29	0.81–2.04	0.85	0.53–1.34	1.26	0.78–2.04
Black	0.71	0.34–1.47	1.59	0.77–3.31	1.38	0.67–2.83	1.60	0.77–3.31	0.46	0.21–1.01	0.52	0.24–1.12
Asian/Pacific Islander	1.26	0.74–2.13	1.75*	1.05–2.93	1.90*	1.13–3.18	1.28	0.77–2.14	1.40	0.84–2.32	2.10**	1.24–3.55
Amer. Indian/multi-racial/other	1.15	0.56–2.36	0.89	0.41–1.90	0.98	0.48–2.00	0.90	0.44–1.83	0.94	0.46–1.91	1.41	0.67–2.94
Language spoken at home												
English (Ref.)	1.00		1.00		1.00		1.00		1.00		1.00	
Non-English	1.14	0.79–1.65	1.64*	1.12–2.39	1.19	0.82–1.73	1.05	0.73–1.52	0.92	0.64–1.32	1.09	0.75–1.58
Food insecure	1.51*	1.05–2.17	1.52*	1.06–2.18	1.13	0.79–1.61	1.43*	1.01–2.04	1.53*	1.08–2.17	2.28***	1.59–3.29
Uninsured	1.21	0.80–1.83	0.84	0.55–1.28	1.11	0.74–1.68	1.59*	1.06–2.39	0.98	0.65–1.47	2.76***	1.82–4.20
No usual source of care	0.86	0.61–1.21	0.90	0.63–1.27	0.71*	0.50–1.00	1.62**	1.15–2.27	1.04	0.74–1.47	1.47*	1.04–2.08
State												
California (Ref.)	1.00		1.00		1.00		1.00		1.00		1.00	
Texas	1.22	0.86–1.73	0.86	0.60–1.24	0.89	0.62–1.27	0.52***	0.37–0.75	0.93	0.65–1.32	1.38	0.96–1.99
History of UTI	0.80	0.56–1.14	0.86	0.59–1.24	0.69*	0.48–0.99	0.98	0.69–1.40	0.81	0.57–1.16	0.95	0.66–1.37

Models included random effects for site

*aOR* adjusted odds ratio, *CI* confidence interval

*p < 0.05; **p < 0.01; ***p < 0.001

Racial and ethnic group was also associated with these barriers. Specifically, compared to Hispanic participants, Asian/Pacific Islander participants had higher odds of concerns about STIs (aOR = 1.75, 95% 1.05–2.93), parental privacy (aOR = 1.90, 95% CI 1.13–3.18), and cost (aOR = 2.10, 95% CI 1.24–3.55). Participants who spoke a language other than English language at home had greater odds of worrying about STIs (aOR = 1.64, 95% CI 1.12–2.39) compared to English-only speakers.

In addition to sociodemographic factors, socioeconomic characteristics were associated with multiple perceived barriers to care for urinary symptoms. Food insecurity was significantly associated with most perceived barriers (aOR ranging from 1.43 to 2.28). Uninsured participants had greater odds of expecting appointment delays (aOR = 1.59, 95% CI 1.06–2.39) and cost concerns (aOR = 2.76, 95% CI 1.82–4.20) compared to those with insurance. Similarly, lacking a usual source of care was associated with expecting appointment delays (aOR = 1.62, 95% CI 1.15–2.27) and cost concerns (aOR = 1.47, 95% CI 1.04–2.08). In sensitivity analyses, the results were consistent when each perceived barrier was modeled as an ordinal outcome.

For the composite score of perceived barriers to UTI care, participants reported an average of 4.18 barriers (SD = 2.96). Table 4 shows the results of the multivariate linear regression model predicting the composite score. Younger participants reported significantly more barriers (*β* = − 0.19, SE = 0.06). Asian/Pacific Islander participants reported more barriers compared to Hispanic participants (*β* = 0.71, SE = 0.35), and participants who spoke a non-English language at home also reported more barriers (*β* = 0.57, SE = 0.25). Additionally, participants experiencing food insecurity (*β* = 1.32, SE = 0.24), those who were uninsured (*β* = 0.65, SE = 0.28), and those without a usual source of care reported significantly more barriers to UTI care (0.97, SE = 0.23). The findings remained consistent when we predicted a composite score calculated by summing the ordinal items.

**Table 4:** Multivariable Mixed-Effects Linear Regression Model Predicting Composite Score of Number of Perceived Barriers to UTI Care (*N* = 667)

	Coefficient	SE
Age (years)	− 0.19***	0.06
Race and ethnicity		
Hispanic	Ref	
White	− 0.28	0.31
Black	0.28	0.50
Asian/Pacific Islander	0.71*	0.35
American Indian/multi-racial/other	0.39	0.49
Language spoken at home		
English	Ref	
Non-English	0.57*	0.25
Food insecure	1.32***	0.24
Uninsured	0.65*	0.28
No usual source of care	0.97***	0.23
State		
California	Ref	
Texas	− 0.03	0.24
History of UTI	− 0.55*	0.24
Intercept	7.35***	1.33

Model also includes random effects for site

*SE* standard error

*p < 0.05; **p < 0.01; ***p < 0.001

## DISCUSSION

UTIs are common, especially among sexually active young women, and are easily treated with antibiotics. Despite this, many participants in this study perceived significant barriers to accessing UTI care. Building on the Levesque framework, these barriers occur at each step of the process patients must navigate, from recognizing the need for care to engaging with healthcare providers. The most common reported barriers included concerns about hearing bad news, worry about potentially having an STI, and fear of parents finding out, highlighting the often sensitive, stigmatized nature of UTIs in this age group. These findings align with qualitative studies conducted in European countries across broader age groups, which have similarly identified concerns about underlying serious illness and fear of judgement, whether internalized or from others.^4^ Cost concerns and perceived delays in appointment scheduling were also important barriers, which are not unique to UTI care in the US healthcare system.^36–38^ Navigating the fragmented and costly healthcare system is especially challenging for emerging adults who must learn to manage it independently, often without health insurance and other resources.

Consistent with the social determinants of health framework and previous research on healthcare access among young adults,^39,40^ our findings highlight significant disparities in UTI care access. Perceived barriers were more prevalent among younger individuals, those identifying as Asian/Pacific Islander, the uninsured, those without a usual source of care, and individuals experiencing food insecurity. Notably, specific barriers varied across groups, with no group consistently reporting all barriers. These results underscore the need for interventions that target multiple barriers and are responsive to the populations they serve.

Our findings highlight the need for improved UTI education, supporting young people to recognize symptoms and seek desired care. Sexual health education should include information about urinary symptoms and their causes—including UTIs and STIs—and UTI diagnosis and management. Research has shown that brochures can significantly improve knowledge about uncomplicated UTIs,^41^ and highly visual, youth-friendly materials can enhance young people’s access to stigmatized health services.^42^ Developing engaging and culturally appropriate UTI educational materials that are tailored to resonate with emerging adults is essential. Effective distribution methods should reach a broad audience of young adults, leveraging social media platforms. While social media use is nearly ubiquitous among young adults,^43^ platforms like YouTube and TikTok often contain misinformation about UTIs.^44^ Addressing this issue with medically accurate, youth-friendly content could inform and support many young people.

Education can also reduce stigma around UTI symptoms, supporting care-seeking. Qualitative studies have found that individuals with UTIs often feel shame or judgement, internalized or a fear of judgement from others,^4^ which can lead to delays in seeking care. Our findings reflect how stigma manifests in perceived barriers to care, including privacy concerns, fear of provider judgement, and discomfort discussing symptoms. School and community-based education can combat stigma by normalizing UTIs and STIs and correcting misconceptions, such as associating them with being unclean or immoral.

Along with education, expanding telehealth could help address barriers to UTI care. Telehealth, the delivery of health services remotely through technology, grew rapidly during the COVID-19 pandemic.^45,46^ Studies have indicated that telehealth management of uncomplicated UTIs can be done safely, effectively, and efficiently.^47–51^ Telehealth visits can help young people overcome barriers like time constraints and difficulty reaching a clinic. Asynchronous telehealth services, such as electronic visits (e-visits) through a secure web-based platform, could be particularly helpful for the nearly half of participants who expected appointment delays, as these services eliminate the need for a scheduled visit.^49,50^ Telehealth also offers greater privacy, allowing young people to feel more comfortable discussing symptoms while engaging in a visit from their own homes. However, studies have shown that adolescents and young adults do not use telehealth regularly and lack the knowledge to access it for services like contraception,^52,53^ a barrier which may also apply for UTI care. Research is needed to address concerns about telehealth for UTI care in young people, including the elevated potential to misdiagnose STIs as UTI.

This study has several strengths and limitations. It addresses a gap in the literature about one of the most common outpatient infections. By applying Levesque’s model of healthcare access and examining a wide range of barriers to UTI care, it provides a comprehensive understanding of access challenges. The diverse sample includes emerging adults recruited from community-based settings; this is an advantage because recruiting participants from clinic-based settings likely excludes individuals experiencing the greatest barriers to care. However, the findings may not be generalizable to all emerging adults in California and Texas or other geographies, as participants were recruited from community colleges. Community college students are less studied than 4-year college students and have unique health needs^54–56^ but limited access to campus health services.^57,58^ In addition, while the study included individuals assigned female at birth of all genders, most participants identified as cisgender, limiting insights into barriers experienced by youth with other gender identities. Small sample sizes for certain subgroups, particularly those who identify as Black, American Indian, or multi-racial, may also limit our ability to detect significant differences. Future research should use larger, representative samples to ensure findings are generalizable to the broader population of emerging adults.

In conclusion, our research revealed substantial challenges to UTI care, especially among younger and socially disadvantaged individuals. Identifying the unique needs of emerging adults is essential for developing interventions to improve their access to UTI care. In turn, improving access can minimize delays in care and reduce pain, suffering, and complications from untreated UTIs, while promoting health equity for UTIs, a significant public health concern in this population. Findings highlighted areas for improvement, including expanding education on UTI diagnosis and management and the overlap in symptoms with STIs, and increasing telehealth options to complement in-person care.

## Data Availability

The dataset analyzed during the current study is available from the corresponding author on reasonable request.

## Declarations:

### Disclaimer:

The funders played no role in the study design, data collection, analysis, interpretation of data, decision to publish, or manuscript preparation.
